# Metabolic Health Is a More Important Determinant for Diabetes Development than Simple Obesity: A 4-Year Retrospective Longitudinal Study

**DOI:** 10.1371/journal.pone.0098369

**Published:** 2014-05-28

**Authors:** Eun-Jung Rhee, Min Kyung Lee, Jong Dae Kim, Won Seon Jeon, Ji Cheol Bae, Se Eun Park, Cheol-Young Park, Ki-Won Oh, Sung-Woo Park, Won-Young Lee

**Affiliations:** 1 Division of Endocrinology and Metabolism, Department of Internal Medicine, Kangbuk Samsung Hospital, Sungkyunkwan University School of Medicine, Seoul, Korea; 2 Division of Endocrinology and Metabolism, Department of Internal Medicine, Samsung Medical Center, Sungkyunkwan University School of Medicine, Seoul, Korea; University of Sao Paulo, Brazil

## Abstract

**Background:**

Recent studies report the importance of metabolic health beyond obesity. The aim of this study is to compare the risk for diabetes development according to different status of metabolic health and obesity over a median follow-up of 48.7 months.

**Methods:**

6,748 non-diabetic subjects (mean age 43 years) were divided into four groups according to the baseline metabolic health and obesity status: metabolically healthy non-obese (MHNO), metabolically healthy obese (MHO), metabolically unhealthy non-obese (MUHNO) and metabolically unhealthy obese (MUHO). Being metabolically healthy was defined by having less than 2 components among the 5 components, that is, high blood pressure, high fasting blood glucose, high triglyceride, low high-density lipoprotein cholesterol and being in the highest decile of homeostasis model assessment-insulin resistance (HOMA-IR) index. Obesity status was assessed by body mass index (BMI) higher than 25 kg/m^2^. The development of diabetes was assessed annually from self-questionnaire, fasting glucose and HbA1c.

**Results:**

At baseline, 45.3% of the subjects were MHNO, 11.3% were MHO, 21.7% were MUHNO, and 21.7% were MUHO. During a median follow-up of 48.7 months, 277 subject (4.1%) developed diabetes. The hazard ratio for diabetes development was 1.338 in MHO group (95% CI 0.67–2.672), 4.321 in MUHNO group (95% CI 2.702–6.910) and 5.994 in MUHO group (95% CI 3.561–10.085) when MHNO group was considered as the reference group. These results were similar after adjustment for the changes of the risk factors during the follow-up period.

**Conclusion:**

The risk for future diabetes development was higher in metabolically unhealthy subgroups compared with those of metabolically healthy subjects regardless of obesity status.

## Introduction

It has been known that adipose tissue is not only a gathering of fat cells, but also an active endocrine organ that secretes various adipocytokines that influences the energy expenditure and metabolism of our body [1]. Furthermore, not the actual amount of adipose tissue, but where they are deposited affects more of their metabolic function; for example, visceral fat is the starting point for the insulin resistance and atherosclerosis as it is more prone to lipolysis and releases free fatty acid to the circulation, whereas subcutaneous fat is known for the protective effect against insulin resistance and obesity [2], [3].

Recently proposed concept of “metabolically healthy obesity” suggests that there is a subset of obese subjects with metabolically healthy phenotype [4]–[6]. These subjects seem to be protected against obesity-induced deterioration of metabolism, such as dyslipidemia, diabetes, hypertension and cardiovascular risk. A recent result from a prospective cohort study of North West Adelade Healthy Study showed that metabolically healthy obese subjects were more likely to develop incident diabetes compared with normal-weight peers [7]. They also reported that the protective phenotype of “healthy obesity” was only seen in certain subset of subjects and not maintained in whole patients.

As the previous study was performed in only Caucasians, we designed this study to compare the risk for diabetes development among the groups divided by baseline metabolic health and obesity status in a median follow-up of 48.7 months in a large cohort of non-diabetic Korean subjects who participated in a health screening program.

## Methods

### Ethics statement

The participants provided their written informed consent for the usage of the health screening data for the research. The design, protocol and the consent procedure of this study were reviewed and approved by Institutional Review Board of Kangbuk Samsung Hospital (KBS12089) and is in accordance with the Helsinki Declaration of 1975.

### Subjects

This was a retrospective study, and subjects were the participants in Kangbuk Samsung Health Study, a large database from the participants in medical health checkup program at the Health Promotion Center of Kangbuk Samsung Hospital, Sungkyunkwan University, Seoul, Korea. The purpose of the medical health checkup program is to promote the health of employees through regular health checkups and to enhance early detection of existing diseases. Most of the examinees are employees and family members of various industrial companies from all around the country. The costs of the medical examinations are largely paid for by their employers, and a considerable proportion of the examinees undergo examinations annually or biannually.

Initial data were obtained from 10,868 participants in whom annual health check-up was performed for five consecutive years between January 2005 and December 2009. Among these, 4,121 subjects were excluded due to presence of diabetes and missing data, especially fasting insulin levels and lipid profiles. Final analyses were performed in 6,748 subjects (4,958 men and 1,790 women) with mean age of 43.0 years.

### Anthropometric and laboratory measurements

Height and weight were measured twice and then averaged. The body mass index (BMI) was calculated by dividing the weight (kg) by the square of the height (m). Blood pressure was measured using a standardized sphygmomanometer after five minutes of rest. The waist circumference (WC) was measured in the standing position, at the middle point between anterior iliac crest and lower border of rib by a single examiner. Values for waist circumference were available only in 2900 subjects due to the inconsistency of measurement method.

Body composition measurements of the subjects were carried out by segmental bioelectric impedance, using eight tractile electrodes according to the manufacturer's instructions (InBody 3·0, Biospace, Korea). Lean mass (kg), fat mass (kg) and per cent fat mass (%) were measured. Skeletal muscle index (SMI) was calculated with the following formula: lean mass (kg)/body weight (kg) ×100 [8].

All of the subjects were examined after an overnight fast. The hexokinase method was used to test fasting glucose concentrations (Hitachi Modular D2400; Roche, Tokyo, Japan). Fasting insulin concentrations were determined by electrochemiluminescence immunoassay (Hitachi Modular E170; Roche, Tokyo, Japan). Alanine aminotransferase (ALT) and aspartate aminotransferase (AST) were measured by UV without the P5P method (Advia 1650 Autoanalyzer, Bayer diagnostics, Leverkusen, Germany). An enzymatic calorimetric test was used to measure the total cholesterol (TC) and triglyceride (TG) concentrations. The selective inhibition method was used to measure the level of high-density lipoprotein cholesterol (HDL-C), and a homogeneous enzymatic calorimetric test was used to measure the level of low-density lipoprotein cholesterol (LDL-C). HbA1c was measured by immunoturbidimetric assay with a Cobra Integra 800 automatic analyzer (Roche Diagnostics, Basel, Switzerland) with a reference value of 4.4–6.4%. The methodology was aligned with the Diabetes Control and Complications Trial (DCCT) and National Glycohemoglobin Standardization Program (NGSP) standards [9]. The intra-assay coefficient of variation (CV) was 2.3% and inter-assay CV was 2.4%, both within the NGSP acceptable limits [10]. Serum high-sensitivity C-reactive protein (hs-CRP) levels were measured by using a nephelometric assay and using a BNII nephelometer (Dade Behring, Deerfield, IL).

The subjects with underlying diabetes at baseline were excluded from the study. The presence of impaired fasting glucose and diabetes mellitus was determined according to the self-questionnaire of the participants and the diagnostic criteria of American Diabetes Association [11]. Development of diabetes was assessed in every year's examination with the same diagnostic criteria of diabetes mellitus.

The presence of hypertension was defined by criteria recommended by the seventh report of the Joint National Committee on prevention, detection, evaluation and treatment of high BP (JNC 7) [12]: ≥140/90 mm Hg or presently taking anti-hypertensive medication.

Insulin resistance was measured using the homeostatic model of the assessment of insulin resistance (HOMA-IR) and was obtained by applying the following formula: HOMA-IR  =  fasting insulin (IU/mL) × fasting blood glucose (mmol/L)/22.5 [13].

A smoker was defined as a subject who had ever smoked at least five total packs of cigarettes in his whole life. Doing regular exercise was defined if the subject does regular exercise of moderate intensity at least three times a week. Alcohol drinking was defined as drinking more than 20 g of alcohol every day. These life style habits were assessed annually by a self-questionnaire.

### Definition of metabolic health and obesity status

Obesity status was defined based on the combined consideration of obesity status by BMI category (non-obese <25 kg/m^2^, obese ≥25 kg/m^2^). In 2000, the WHO Western Pacific Region suggested revised Asia–Pacific criteria of obesity in Asian populations using reduced values for body mass index (BMI)≥25 kg/m^2^ in both sexes [14].

Being metabolically healthy was defined by having less than two metabolic abnormalities among the four components of metabolic syndrome besides WC criteria plus insulin resistance status defined by HOMA-IR, which was modified from the criteria by Wildman et al. [15], [16]:

1) Systolic blood pressure≥130 mmHg and/or diastolic blood pressure ≥85 mmHg or on antihypertensive treatment

2) Triglyceride ≥150 mg/dL

3) Fasting glucose ≥100 mg/dL

4) HDL-cholesterol <40 mg/dL in men, <50 mg/dL in women

5) HOMA-IR ≥90th percentile

According to the above criteria, participants were divided into 4 groups:

1) Metabolically healthy, non-obese (MHNO): BMI <25 kg/m^2^ and <2 metabolic risk factor

2) Metabolically healthy, obese (MHO): BMI ≥25 kg/m^2^ and <2 metabolic risk factor

3) Metabolically unhealthy, non-obese (MUHNO): BMI <25 kg/m^2^ and ≥2 metabolic risk factor

4) Metabolically unhealthy, obese (MUHO): BMI ≥25 kg/m^2^ and ≥2 metabolic risk factor

### Statistical analysis

All data were analyzed using SPSS Windows version 18.0 (SPSS Inc., Chicago, IL, USA). Comparisons of the mean values and the prevalence of metabolic variables among the four groups divided by baseline metabolic health and obesity status were performed with one-way analysis of variance (ANOVA) test and chi-square test, and data that do not follow normal distribution were analyzed after logarithmic transformation. Comparisons of hazard ratio (HR) for incident diabetes development in four groups divided by baseline metabolic health and obesity were analyzed with cox proportional hazard model analyses after adjustment for confounding variables at baseline and the changes of the risk factors, such as, body weight, alcohol drinking status, medication, smoking and regular exercise, from the baseline period to the time point of diabetes development. Kaplan-Meier survival analyses were performed with incident diabetes development after 4 years according to the baseline metabolic health and obesity status. Statistical significance was defined as *p*<0.05.

## Results

### Study population

Mean age of the total participants was 43 years (Table 1). At baseline, 3055 (45.3%) subjects were in MHNO group, 762 (11.3%) subjects in MHO group, 1464 (21.7%) subjects in MUHNO group, and 1467 (21.7%) subjects in MUHO group. Among the subjects, 1,845 subjects (27.3%) were in impaired fasting glucose (IFG) status, and the proportion of subjects who were in IFG status was higher in metabolically unhealthy groups compared with metabolically healthy groups.

**Table 1:** Comparison of baseline characteristics between the groups divided by metabolic health and obesity status.

N = 6,748 (%)	Total	MHNO	MHO	MUHNO	MUHO	*P value* *
		N = 3055 (45.3)	N = 762 (11.3)	N = 1464 (21.7)	N = 1467 (21.7)	
Age (years)	43.0±4.8	42.8±4.9^a^	43.1±4.6^a,b,c^	43.4±4.9^b,d^	43.1±4.5^c,d^	<0.01
Gender: men (%)	4958 (73.5)	1752 (57.3)	599 (78.6)	1235 (84.4)	1372 (93.5)	<0.01
FBS (mg/dL)	95.3±8.5	91.9±6.9	93.6±6.4	98.5±8.7	100.1±8.8	<0.01
AST (IU/L)	23.8±8.8	21.9±7.0	24.3±8.4	23.8±9.2	27.2±10.7	<0.01
ALT (IU/L)	26.0±17.5	20.0±11.3	27.7±15.7^a^	27.4±19.9^a^	36.3±20.9	<0.01
BUN (mg/dL)	14.0±3.3	13.7±3.3	14.7±3.5^a^	14.0±3.3	14.5±3.4^a^	<0.01
Serum creatinine (mg/dL)	1.1±0.2	1.02±0.15	1.09±0.14	1.10±0.14	1.14±0.14	<0.01
TC (mg/dL)	193.7±32.2	187.3±30.6	198.7±29.9	195.4±32.5	202.9±33.5	<0.01
Triglyceride (mg/dL)	137.0±84.9	93.6±36.3	114.0±44.0	177.5±94.0	198.8±104.7	<0.01
HDL-C (mg/dL)	51.4±11.6	56.7±11.7	53.1±0.2	46.3±9.4	44.8±8.0	<0.01
LDL-C (mg/dL)	112.4±27.0	107.5±25.9	119.8±25.3^a^	112.6±26.9	118.7±27.8^a^	<0.01
HbA1c	5.37±0.3	5.33±0.3	5.36±0.3^a^	5.39±0.4^a^	5.46±0.4	<0.01
Fasting insulin (IU/L)	8.6±3.2	7.3±2.3	8.6±2.6^a^	8.8±3.1^a^	10.9±3.9	<0.01
Hs-CRP (mg/dL)	0.12±0.3	0.09±0.3	0.13±0.3	0.11±0.2	0.16±0.4	<0.01
Body weight (kg)	67.7±10.8	60.8±8.2	75.1±7.4	66.7±7.2	79.0±7.6	<0.01
BMI (kg/m^2^)	23.9±2.8	22.0±1.8	26.5±1.2	23.2±1.4	27.2±1.8	<0.01
Systolic BP (mmHg)	113.6±14.3	107.9±11.8	112.6±11.3	118.0±14.6	121.8±14.8	<0.01
Diastolic BP (mmHg)	75.8±10.0	71.6±8.2	75.6±8.1	78.5±9.7	81.9±10.4	<0.01
Waist circumference (cm)†	80.9±9.0	75.2±7.4	87.7±5.9	80.9±6.1	90.0±5.6	<0.01
Lean mass (kg)	48.8±8.3	44.6±7.6	52.4±7.4	49.0±6.7	55.2±6.2	<0.01
Skeletal muscle index	72.1±5.1	73.1±5.3	69.5±5.1^a^	73.4±4.3	69.9±4.1^a^	<0.01
Body fat mass (kg)	16.1±4.6	13.6±3.3	19.7±3.5	14.8±2.8	20.7±4.0	<0.01
Percent body fat (%)	23.7±5.4	22.5±5.5^a^	26.5±5.3^b^	22.4±4.5^a^	26.2±4.3^b^	<0.01
HOMA-IR	2.0±0.8	1.67±0.5	1.99±0.6	2.15±0.8	2.71±1.0	<0.01
Subjects with IFG (%)	1845 (27.3)	301 (9.9)	79 (10.4)	712 (48.6)	753 (51.3)	<0.01
Diabetes development (%)	277 (4.1)	27 (0.9)	14 (1.8)	79 (5.4)	157 (10.7)	<0.01
Subjects who have ever smoked (%)‡	3632 (53.8)	1242 (41.4)	433 (57.7)	923 (63.7)	1034 (71.2)	<0.01
Alcohol drinking (%)	599 (8.9)	215 (7.0)	82 (10.8)	148 (10.1)	154 (10.5)	<0.01
Regular exercise (%)	1246 (18.5)	645 (21.1)	158 (20.7)	217 (14.8)	226 (15.4)	<0.01
Antihypertensive medication (%)	353 (5.2)	51 (1.7)	19 (2.5)	103 (7.0)	180 (12.3)	<0.01
Education status (%)						0.056
No education	14 (0.2)	10 (0.3)	0 (0)	2 (0.1)	2 (0.1)	
Elementary school	117 (1.7)	54 (1.8)	13 (1.7)	28 (1.9)	22 (1.5)	
Middle school	66 (1.0)	34 (1.1)	12 (1.6)	11 (0.8)	9 (0.6)	
High school	1588 (23.5)	771 (25.2)	166 (21.8)	343 (23.4)	308 (21.0)	
Technical college	573 (8.5)	260 (8.5)	63 (8.3)	120 (8.2)	130 (8.9)	
High than university	4390 (65.1)	1926 (63)	508 (66.7)	960 (65.6)	996 (67.9)	

MHNO, metabolically healthy non-obese; MHO, metabolically healthy obese; MUHNO, metabolically unhealthy non-obese; MUHO, metabolically unhealthy obese; FBS, fasting blood sugar; AST, Aspartate aminotransferase; ALT, Alanine aminotransferase; BUN, blood urea nitrogen; TC, total cholesterol; HDL-C, high-density lipoprotein cholesterol; LDL-C, low-density lipoprotein cholesterol; HbA1c, glycosylated hemoglobin; hs-CRP, high-sensitivity C-reactive protein; BMI, body mass index; BP, blood pressure; HOMA-IR, homeostasis model assessment index - insulin resistance, IFG, impaired fasting glucose.

^*^ *P* values for one-way ANOVA test. ^a,b,c,d,e^ Same letters denote no significant differences between the designated groups in post-hoc analyses. Otherwise, groups showed significant differences between each groups with post-hoc analyses.

† Values for waist circumference were available only in 2900 subjects due to the inconsistency in measurement method.

‡ Subjects who have ever smoked more than 5 packs of cigarettes.

### Comparison of the baseline characteristics of the participants in groups divided by metabolic health and obesity status

Metabolically unhealthy groups showed significantly worse mean values in FBS, serum creatinine, TG, HDL-C, fasting insulin, BP and HOMA-IR compared with metabolically healthy groups. Obese groups showed significantly worse mean values in liver enzymes, total cholesterol, body weight and BMI compared with non-obese subjects (Table 1). High-sensitivity C-reactive protein (hs-CRP) showed the highest mean value in MUHO group among the four groups, and MHO group showed significantly lower mean value of hs-CRP compared with MUHO group. Although waist circumference (WC) values were available only in 2,900 participants due to the inaccuracy in measurement method, metabolically unhealthy subjects showed significantly larger WC compared with metabolically healthy obese or non-obese peers, although there were no differences in percent body fat between obese or non-obese peer groups. Obese groups showed significantly lower skeletal muscle index (SMI) compared with non-obese groups.

Significantly more subjects have ever smoked more than five packs of cigarettes and lesser subjects exercised regularly in metabolically unhealthy groups compared with metabolically healthy groups. MUHNO group showed the lowest proportion of subjects who exercised regularly. Obese subjects tended to drink more alcohol compared with non-obese subjects (Table 1).

**Figure 1:**
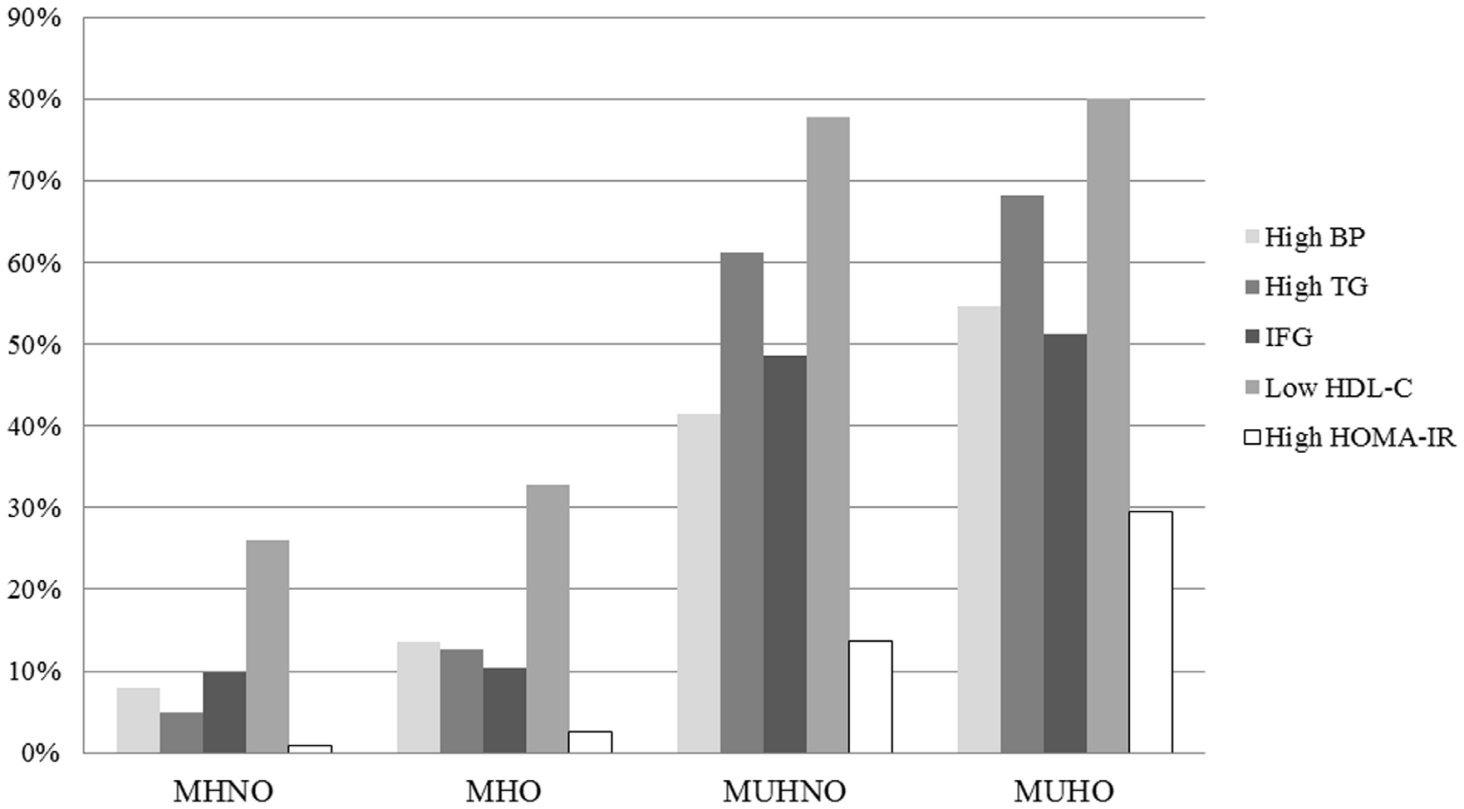
Comparisons of prevalence of metabolic components according to the four groups divided by baseline metabolic health and obesity status.

When the prevalence of metabolic components that were included in the assessment of metabolic health were compared among the groups divided by baseline metabolic health and obesity status, the prevalences for all the components were higher in the metabolically unhealthy groups (MUHNO and MUHO) compared with those in the metabolically healthy groups (MHNO and MHO) (Table 2, Figure 1). Among the components, the prevalences for hypertriglyceridemia and low HDL-C were markedly higher in metabolically unhealthy groups compared to other components in metabolically healthy groups.

**Table 2:** Comparisons of baseline prevalence of metabolic components between the groups divided by metabolic health and obesity status.

	Total	MHNO	MHO	MUHNO	MUHO	*P value*
		N = 3055	N = 762	N = 1464	N = 1467	
High blood pressure	1758 (26.1)	244 (8.0)	104 (13.6)	608 (41.5)	802 (54.7)	<0.01
High TG	2147 (31.8)	152 (5.0)	96 (12.6)	898 (61.3)	1001 (68.2)	<0.01
Low HDL-C	3352 (49.7)	793 (26.0)	249 (32.7)	1137 (77.7)	1173 (80.0)	<0.01
IFG	1845 (27.3)	301 (9.9)	79 (10.4)	712 (48.6)	753 (51.3)	<0.01
High HOMA-IR	675 (10.0)	24 (0.8)	19 (2.5)	199 (13.6)	433 (29.5)	<0.01

MHNO, metabolically healthy non-obese; MHO, metabolically healthy obese; MUHNO, metabolically unhealthy non-obese; MUHO, metabolically unhealthy obese; TG, triglyceride; HDL-C, high-density lipoprotein cholesterol; IFG, impaired fasting glucose; HOMA-IR, homeostasis model assessment index - insulin resistance.

### Comparison of the risk and the development rate of diabetes in groups divided by baseline metabolic health and obesity status

During a median follow-up of 48.7 months, 277 subjects (4.1%) developed diabetes. MUHO group showed the highest rate for incident diabetes by 10.7% and MHNO group showed the lowest rate for incident diabetes by 0.9% among the four groups (Table 1). MHO subjects showed lower rate for incident diabetes development (1.8%) compared with MUHNO subjects (5.4%).

**Figure 2:**
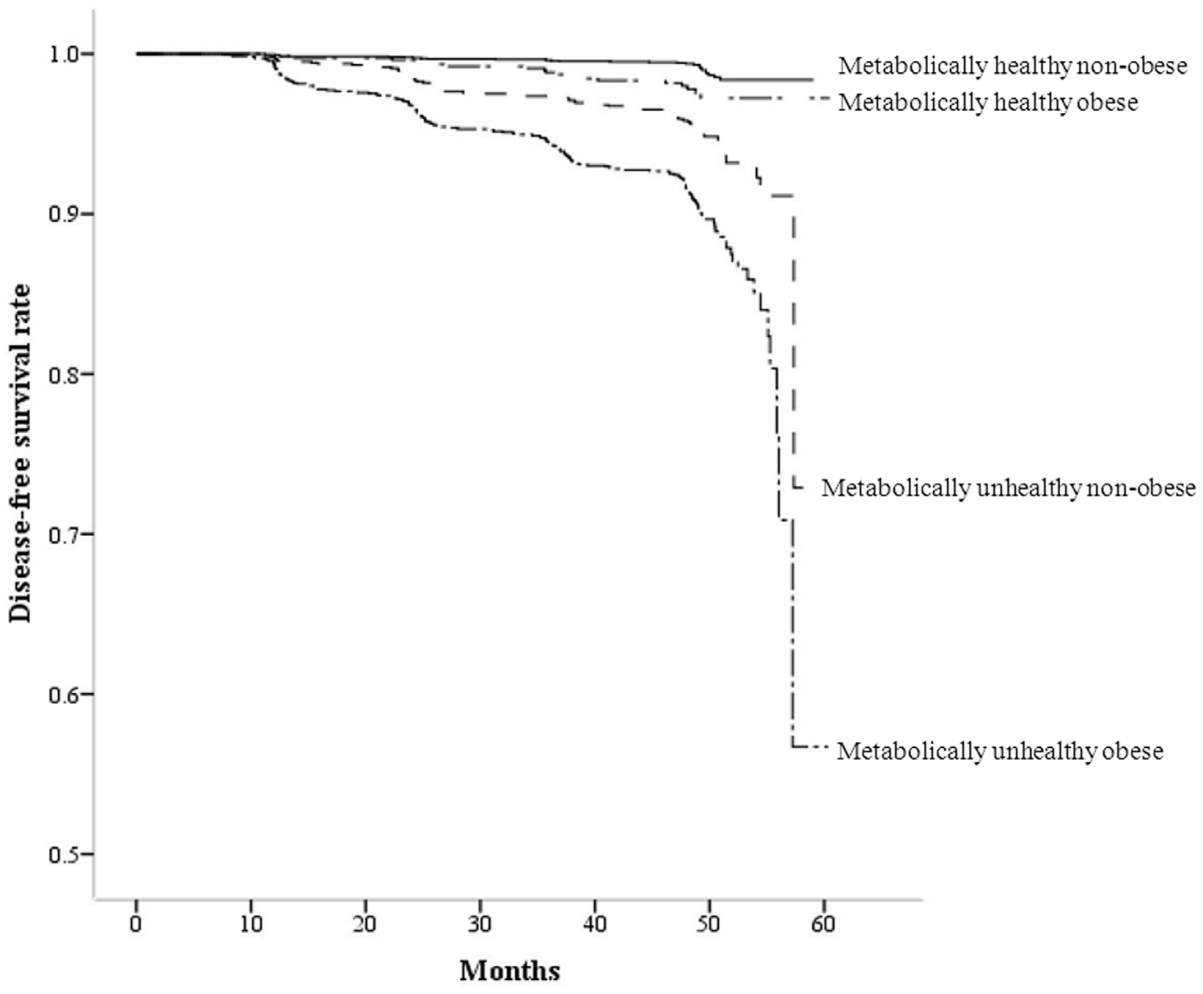
Disease-free survival by Kaplan-Meier analysis. Median follow-up period was 48.7 months. Subjects were divided into four groups according to baseline metabolic health and obesity status.

In a cox-proportional hazard model with diabetes development as the dependent variable, MHO subjects showed HR of 1.338, MUHNO subjects showed HR of 4.321 and MUHO subjects showed HR of 5.994 for diabetes development after adjustment for baseline confounding factors with MHNO group as the reference group (Table 3). Similar results were observed when changes in the confounding factors during the follow-up period, such as, changes in body weight, exercise status, alcohol drinking and antihypertensive medication, were included in the same model. In Kaplan-Meier disease-free survival analysis, MUHO group showed the lowest disease-free survival for diabetes among the four groups, and MUHNO group showed the second lowest disease-free survival next to MUHO group (Figure 2). MHO group showed higher disease-free survival compared to metabolically unhealthy groups.

**Table 3:** Hazard ratio for incident diabetes according to baseline metabolic health and obesity status.

	Hazard ratio	95% confidence interval		*P value*
		Upper	Lower	
Model 1*				
Age	1.034	1.012	1.058	0.003
Gender	0.744	0.393	1.408	0.363
Alanine aminotransferase	1.008	1.005	1.011	<0.01
Serum creatinine	0.663	0.216	2.039	0.474
Total cholesterol	1.006	1.002	1.009	0.001
High-sensitivity C-reactive protein	1.116	0.902	1.381	0.311
Skeletal muscle index	1.919	0.561	6.569	0.299
Percent body fat	1.934	0.599	6.238	0.270
Systolic blood pressure	1.004	0.995	1.012	0.387
Groups by metabolic health and obesity				
MHNO	1.000	-	-	<0.01
MHO	1.338	0.670	2.672	0.410
MUHNO	4.321	2.702	6.910	<0.01
MUHO	5.994	3.561	10.085	<0.01
Model 2†				
Age	1.040	1.017	1.064	0.001
Gender	0.694	0.367	1.311	0.260
Alanine aminotransferase	1.009	1.006	1.012	<0.01
Serum creatinine	0.564	0.181	1.756	0.323
Total cholesterol	1.006	1.002	1.009	0.001
High sensitivity C-reactive protein	1.143	0.911	1.435	0.248
Skeletal muscle index	2.157	0.642	7.248	0.214
Percent body fat	2.167	0.684	6.862	0.189
Systolic blood pressure	1.007	0.998	1.016	0.111
Groups by metabolic health and obesity status				
MHNO	1.000	-	-	<0.01
MHO	1.385	0.694	2.765	0.356
MUHNO	4.458	2.790	7.122	<0.01
MUHO	6.489	3.873	10.871	<0.01

MHNO, metabolically healthy non-obese; MHO, metabolically healthy obese; MUHNO, metabolically unhealthy non-obese; MUHO, metabolically unhealthy obese.

^*^ Additional adjustment for baseline history of smoking, alcohol drinking, regular exercise status, education and antihypertensive medication.

† Additional adjustment for baseline history of smoking and education, and changes of body weight, alcohol drinking, regular exercise status and antihypertensive medication during the follow-up period.

## Discussion

In this study, metabolically unhealthy subjects showed significantly increased risk for diabetes development in a median 48.7 months of follow-up period compared with metabolically healthy subjects, regardless of obesity status assessed by BMI. In a cox-proportional hazard model, MUHNO and MUHO groups showed significantly higher HR for diabetes development with over four-fold increased risk for diabetes compared with MHNO group. These results were similar after adjustment for changes in the confounding factors during the follow-up period. In addition, MUHO group showed larger mean value for WC compared with that of MHO group with similar amount of percent body fat, suggesting the role of visceral obesity on deterioration of metabolic health. These findings suggest the importance of metabolic health in the development of diabetes and further importance of visceral obesity in metabolic health apart from simple obesity assessed by BMI larger than 25 kg/m^2^ in this study population.

There are not many studies reported on the relationship between diabetes development and metabolic healthy obesity. In a very recent prospective study performed in 3,743 Caucasians who were followed up for 5.5–10.3 years, MHO subjects were more likely to develop metabolic risk and incident diabetes compared with metabolically healthy normal-weight subjects [7]. This is in line with our study result in that metabolically unhealthy subjects showed higher risk for diabetes development compared with metabolically healthy subjects by more than three-fold even after adjustment for possible confounding factors at baseline and during the follow-up period, suggesting the importance of metabolic health assessed by various metabolic risk factors compared with simple obesity derived from BMI. At least, it is apparent from the findings of our study that simple obesity assessed by BMI is not the form of obesity that could affect metabolic health and subsequent development of diabetes.

Then, what is the factor that cause differences in the outcome between metabolically healthy an unhealthy obese- or non-obese subjects? The results from previous studies suggest the differences in the body composition, physical activity, cardio-respiratory fitness, different adipocyte characteristics and the amount of inflammatory response as the mechanisms that underlie the distinction between metabolically healthy and unhealthy obese subjects [4], [17]–[19]. Apart from already known assumed mechanisms, a very recent study performed in 2,047 Caucasians [20] reported that moderate and high levels of physical activity and compliance with food pyramid recommendations increased the likelihood of MHO.

In our study, metabolically unhealthy groups tended to exercise less and had higher prevalence for smoking experience compared with metabolically unhealthy groups. Especially, MUHNO group showed the lowest proportion of subjects who exercised regularly among the four groups. In addition, mean WC was larger in metabolically unhealthy subjects compared with metabolically healthy obese or non-obese peers, with the similar percentage of body fat, suggesting the importance of not just the amount of fat, but where the fat is accumulated. Another important difference between metabolically healthy and unhealthy groups was markedly higher prevalences for atherogenic dyslipidemia represented by hypertriglyceridemia and low HDL-C compared to other components such as, IFG, high blood pressure or insulin resistance, suggesting the relatively stronger effects of dyslipidemia on the development of diabetes even among the metabolic parameters. This is in line with previous study that reported the independent association of atherogenic lipoprotein abnormality with development of type 2 diabetes [21]. In addition, metabolically unhealthy groups showed significantly higher level of hs-CRP compared with metabolically healthy peers, suggesting the role of systemic inflammation in the development of diabetes. These results suggest the importance of life style modification and subsequent reduction in visceral obesity and systemic inflammation in maintaining metabolic health and normoglycemia, not just the reduction of body weight *per se*.

Our study has limitations. As our study is observational, the precise mechanism for the results could not be fully explained. The lack of post-challenge glucose level in the diagnosis of diabetes could have biased the true proportion of diabetes patients. However, as we included HbA1c higher than 6.5% and medication history of diabetes in the definition of diabetes, this could have sufficient power to exclude subjects with diabetes [22]. Second, as most of the participants were only slightly obese Asians (BMI <30 kg/m^2^), there are limitations on the application of our results in subjects with higher grades of obesity. Thirdly, we used 5 components, that is, hypertension, IFG, hypertriglyceridemia, low HDL-C and being in the highest decile of HOMA-IR, for the assessment of metabolic health instead of 6 components used in the previous studies [15], [23]. However, we were careful about including or using a certain cutoff of hs-CRO for the definition of metabolic health in this population, since levels of hs-CRP might be much different among different ethnic groups [24]. Fourth, we could not adjust for family history of type 2 diabetes of the participants in cox-proportional hazard model. Heritability of type 2 diabetes is estimated at 22∼73% from twin and family studies, although multiple genetic and environmental factors influence the development of diabetes in real practical setting [25]–[27]. Lastly, since there is no unified definition of metabolic health, there might be considerable differences in the outcomes according to which definition is applied [28]. With all these limitations, this study is meaningful in that it was the first study performed in Asian population regarding this issue.

In conclusion, the risk for future diabetes development was higher in metabolically unhealthy subjects compared with those of metabolically healthy subjects regardless of obesity status. Therefore, being metabolically unhealthy might be more important for the development of diabetes than simply being obese. We should make efforts to reverse the metabolic health in these high-risk subjects, and to initiate early intensive life-style modification in these subjects, as simple weight loss might not be the optimal solution for them.
